# Changes in cognitive functioning and quality of life after relocation to a nursing home: a prospective longitudinal study of Swiss nursing home residents

**DOI:** 10.1007/s10433-025-00869-7

**Published:** 2025-07-22

**Authors:** Emmie A. M. Verspeek, Maximilian Haas, Yvonne Brehmer, Manon A. van Scheppingen, Nadine Bender, Matthias Kliegel, Alexandra Hering

**Affiliations:** 1 Department of Developmental Psychology, Tilburg University, Tilburg, Netherlands; 2 Faculty of Psychology and Educational Sciences, University of Geneva, 40, Boulevard du Pont-d’-Arve, 1211 Geneva, Switzerland; 3 Faculty of Psychology, UniDistance Suisse, Brig, Switzerland; 4 Aging Research Center, Karolinska Institutet, Stockholm, Sweden; 5 Centre for the Interdisciplinary Study of Gerontology and Vulnerability, University of Geneva, Geneva, Switzerland; 6 LIVES, Overcoming Vulnerability: Life Course Perspective, Swiss National Centre of Competence in Research, Geneva, Switzerland

**Keywords:** Life transitions, Relocation, Older adults, Cognitive functioning, Quality of life

## Abstract

A relocation to a nursing home is a major life transition in older age, with potential impacts on cognitive functioning and quality of life. In line with psychosocial models of successful aging, we investigated how older adults adapted to this major life transition. Using data collected at nine nursing homes in Geneva, Switzerland, we studied changes in cognitive functioning (i.e., short-term, long-term, and working memory, verbal fluency, prospective memory, and inductive reasoning) and quality of life (i.e., emotional and social loneliness, depressive symptoms, and purpose in life) in cognitively healthy older adults. Moreover, we exploratorily studied whether relational resources (e.g., participation in activities) impacted these changes. Forty-seven nursing home residents, aged 59 to 99 years (*M* = 85.55, *SD* = 9.43, 36 women), completed a neuropsychological test battery and self-report questionnaires approximately one, three, and six months after relocation. Repeated Measures ANOVAs indicated that short-term and long-term memory declined and (emotional) loneliness increased during the first three months after relocation. Yet, no differences were found between the first and last assessment of those indicators. Other factors did not show significant changes over time. Exploratory analyses revealed that visits of friends and participation in activities may play an important role in the impact of- and adaptation to this life transition. Although our results suggest preserved psychosocial adaptational capacities in nursing home residents, we should remain vigilant for the potential negative short-term and long-term impacts of major life transitions in this vulnerable population of older adults.

**Supplementary Information:**

The online version contains supplementary material available at 10.1007/s10433-025-00869-7.

## Introduction

With advancing older age, the ratio of developmental gains and losses becomes increasingly negative (Baltes and Baltes 1990; Freund and Riediger 2003). Older adults generally experience declines in physical and cognitive functioning, and may eventually have to relocate to a nursing home (Gaugler et al. 2007). Relocation to a nursing home is considered a major life transition in older age (Fitzpatrick and Tzouvara 2018) and, potentially has substantial negative impacts on quality of life and cognitive functioning (Franks et al. 2021; Luhmann et al. 2012). 

Psychosocial models of successful aging describe the adaptive capacities of older adults to age successfully, even when losses predominate gains (Baltes and Baltes 1990; Freund and Baltes 2000, 2002). These adaptive capacities partially depend on the resources acquired, maintained, or lost throughout life. Specifically, relational resources (e.g., friends or social engagement) are essential as social connectivity may promote psychological well-being or increase resilience against potential negative impacts of major life events (Cullati et al. 2018). Relational resources may serve a protective role, specifically when connections with family or friends are maintained, or new connections are formed in the nursing home (Baltes et al. 1991; Cooney 2012; Owen et al. 2023). However, older adults may be particularly vulnerable when they did not have relational resources before and/or fail to re-establish them after this major life transition. Hence, whether and how older adults are impacted by relocation to a nursing home may depend on individuals’ relational resources.

The present study examines adaptational processes in a highly vulnerable population of recently relocated nursing home residents. Using a prospective longitudinal design, we investigated short-term changes in the domains of cognitive functioning and quality of life from the first month after relocation (T1) to three months (T2) and six months follow-up (T3). Additionally, we extended previous research by exploring the impact of relational resources on how nursing home residents change in cognitive functioning and quality of life after this major life transition. 

## Changes in cognitive functioning

In general, studies reported large age-related declines in cognitive functioning, as part of the normal aging process (Tucker-Drob et al. 2019). In addition, cognitive abilities are potentially negatively impacted by major life events (Franks et al. 2021). Individuals who experienced major life events (e.g., loss of a (grand)child, close relative, or partner, illness of a partner) performed worse in terms of global cognitive functioning, processing speed, and memory and also showed accelerated declines in their cognitive abilities (Aartsen et al. 2005; Comijs et al. 2011).

Cognitive declines have also been related to the specific major life event of relocating to a nursing home (Scocco et al. 2006) and were greater compared to older adults who remained community-dwelling across a 22-year follow-up (Harmand et al. 2014). Aforementioned studies had a minimum timespan of six months between assessments (i.e., Scocco et al. 2006) and included long-term follow-up assessments of three years (i.e., Aartsen et al. 2005; Comijs et al. 2011). Our study will contribute to the literature by focusing on short-term changes in cognitive functioning after the major life transition of a relocation to a nursing home, a time-frame that has not yet received much scientific attention.

## Changes in quality of life

Another factor potentially impacted by major life events is quality of life (Lucas 2007; Luhmann et al. 2012), which includes evaluations of physical health, social relationships, psychological well-being, living conditions, and personal fulfilment (World Health Organization 1997, 2006). Relocation to a nursing home is often prompted by common challenges in older age such as health events or functional and cognitive declines (Gaugler et al. 2007). Declines or losses in these domains potentially hinder attaining goals in previously familiar activities (Bondevik and Skogstad 2000; Pfund and Lewis 2020). In fact, older adults seem to experience more depressive symptoms from the first week up to six months after relocating to a nursing home (Scocco et al. 2006). In addition, qualitative studies have outlined experiencing disruptions in (and even losses of) routines, relationships, and the sense of belonging as processes that relate to the potential negative impact of relocating to nursing homes on quality of life (Cooney 2012; Johnson and Bibbo 2014; Owen et al. 2023). Yet, more quantitative evidence on changes in depressive symptoms, loneliness, and purpose in life is lacking, which is the focus of the present study.

## Individual differences in adaptation: importance of relational resources

Whether and how older adults are impacted by the relocation to a nursing home may depend on relational resources, and more specifically whether or not individuals are able to maintain or re-establish connectivity across this transition. Relational resources, including friendships or leisure time activities, play a pivotal role in psychological well-being and resilience in response to major life events (Cullati et al. 2018). Moreover, these resources are important for cognitive functioning (Kelly et al. 2017), and various indicators of quality of life (Cooney et al. 2014).

Nursing home residents have previously been found to possess the adaptive capacities to maintain connectivity to friends or family, and to re-establish connectedness (e.g., forming new friendships; Baltes et al. 1991) and a sense of belonging over time (Cooney 2012; Owen et al. 2023). Specifically, older adults who lose connectivity to former relations and/or fail to re-establish social connectivity in the nursing home environment may struggle to adapt to the transition of relocation to a nursing home. Since previous research involved mostly older adults who had lived in a nursing home for a few years (e.g., Owen et al. 2023), the role of relational resources in adaptational processes within the first six months after relocating to a nursing home remains unclear.

## Present study

In this study, we investigated the impact of the major life event of relocation to a nursing home by assessing changes in cognitive abilities and quality of life in the first (T1), three (T2) and six months (T3) after relocation. Based on previous empirical studies (Harmand et al. 2014; Scocco et al. 2006), we expected cognitive abilities to decline after older adults relocated to a nursing home (*Hypothesis 1*). Based on previous quantitative (Scocco et al. 2006) and qualitative studies (Cooney et al. 2014; Johnson and Bibbo 2014; Owen et al. 2023), we hypothesized that older adults experience a decrease in quality of life after relocation to a nursing home (*Hypothesis 2*), translating to increases in feelings of depressive symptoms and loneliness, and decreases in sense of purpose in life. Since little is known about the quantitative short-term changes in terms of cognitive abilities and quality of life, we have drawn up general hypotheses without specification of the timeline of those changes. In addition, previous studies showed the importance of relational resources for the maintenance of cognitive abilities (Kelly et al. 2017) as well as quality of life (Cooney et al. 2014). Hence, in exploratory analyses, we further examined whether the observed changes in cognitive functioning and quality of life were impacted by older adults’ relational resources, focusing on visits by children and friends, participation in activities, and feeling integrated or at home.

Taken together, we aim to provide insights into the short-term impacts of a relocation to a nursing home, as well as potential influential factors that may facilitate adaptational processes after this transition. As such, we aim to inform theories on successful aging and the underlying adaptive capacities of older adults in response to this major life event.

## Methods

### Participants

A total of 110 older adults who recently relocated to nursing homes were contacted to participate in the study between May 2016 and April 2017 in Geneva, Switzerland. Our final sample included 47 individuals (36 women) with complete information at all three assessments. Participants were aged 59 to 99 years (*M* = 85.55, *SD* = 9.43), had an average of 11.09 years of education (*SD* = 3.59), and did not show any signs of severe cognitive impairment at the initial screening assessment using the F-TICS-m described below (*M* = 31.98, *SD* = 3.94; range 28–41). Twenty-two participants were widowed, seven married, and 18 separated, divorced, or single at the first assessment. Supplementary Information S1 provides more information on reasons for attrition and sample selection.

### Material

#### Cognitive functioning

Changes in cognitive abilities were assessed using three parallel versions of the Cognitive Telephone Screening Instrument (Cog-Tel; for full details on the tests see: Kliegel et al. 2007). The Cog-Tel assesses short- and long-term memory, working memory, inductive reasoning, verbal fluency, and prospective memory. Analyses were conducted for the six sub-domains individually, as well as for the combined weighted total score of cognitive functioning derived from the subtests (for full details see: Breitling et al. 2010). The Cog-Tel instrument shows high convergent validity with the Mini Mental State Examination and is sensitive to detecting meaningful short-term cognitive changes, indicated by high test–retest reliability (Ihle et al. 2017).

##### Short-term and long-term memory

Episodic verbal memory was assessed by immediate and delayed recall of eight word pairs, respectively. Half of the pairs were semantically associated (e.g., fruit-apple), whereas the other half of the word pairs were not semantically associated (e.g., salad-pen). The total scores corresponded to the number of correctly recalled word pairs (0 to 8).

##### Working memory

The working memory subtest required participants to repeat a series of numbers in reverse order. Series started with two digits and increased by one digit after every two series up to a total of six different digits. Scores corresponded with the total number of correctly repeated series (0 to 12).

##### Verbal fluency

To assess their verbal fluency, participants were asked to name as many words as possible within one minute using both a semantic (i.e., words starting with a specific letter such as ‘A’ or ‘S’) and a categorical condition (i.e., words corresponding to a specific category such as ‘animals’). For the present analyses, the total verbal fluency score reflected the sum of all correct words listed in both conditions.

##### Inductive reasoning

Up to eight series of five digits each were presented to participants for which they should deduce the underlying rule and identify the sixth digit that would logically complete the series. The test ended after two consecutive errors. The total score on inductive reasoning reflected the number of sequences correctly completed (0 to 8).

##### Prospective memory

Prospective memory was assessed using an event-based task inspired by the Rivermead Behavioral Memory Test (Wilson et al. 1985). At the beginning of the Cog-Tel, participants were instructed to state their year of birth when they were told to name words belonging to a specific category at a later time during the test. Prospective memory was scored depending on whether or not the participant remembered the task (0 versus 1). As participants showed overall high performance across all time points (i.e., limited variability), no individual analyses were conducted on this subdomain and prospective memory was only included in the total cognition score.

#### Quality of life

##### Depressive symptoms

Current depressive symptoms were evaluated using the French version of the Geriatric Depression Scale (GDS-15; Sheikh and Yesavage 1986). Reflecting on the past few weeks, participants were asked about depressive symptoms (e.g., “Do you feel that your life is empty?”). Sum scores were calculated, with higher scores indicating more depressive symptoms. Cronbach’s alpha ranged from 0.740 to 0.781 across the three assessments.

##### Loneliness

For the purpose of this study, the Loneliness Scale (De Jong Gierveld and van Tilburg 1999), was translated into French by a member of the research team. The scale assessed two dimensions of loneliness, tapping into social (e.g., “There are plenty of people I can lean on when I have problems”) and emotional experiences (e.g., “I experience a general sense of emptiness”). These items were answered on a three-point scale: 0 = *no*, 1 = *more or less*, 2 = *yes*. The items referring to the social dimension of loneliness were reverse coded; mean scores for the subscales and the total scale were calculated, with higher scores reflecting greater loneliness. For the subscales of social and emotional loneliness, Cronbach’s alpha ranged from 0.662 to 0.739 and from 0.655 to 0.814, respectively. For the total scale, Cronbach’s alpha values ranged from 0.737 to 0.802 across the three assessments.

##### Purpose in life

Purpose in life was assessed with the 14-item subscale of the Psychological Wellbeing scale (Ryff 1989; Ryff and Keyes 1995; translated by: Lapierre and Desrochers 1997). Participants were asked to indicate whether statements (e.g., “I have a sense of direction and purpose in my life”) applied to their situation on a five-point Likert scale (0 = *strongly disagree*, 4 = *strongly agree*). Mean scores were calculated, with higher scores reflecting higher purpose in life. Cronbach’s alpha values ranged from 0.662 to 0.765,.

#### Indicators of relational resources

At all assessments, respondents answered an anamnestic questionnaire, which included questions about their relational resources. At the first assessment, nursing home residents were asked whether children or friends visited them, and whether they participated in activities in the nursing home (0 = *no*, 1 = *yes*). At the second and third assessments, questions included whether or not participants felt integrated or at home, and whether they participated in activities (0 = *no*, 1 = *yes*).

### Procedure

Individuals residing at nine nursing homes in the canton Geneva, Switzerland, participated in the study. The majority of participants in this sample resided in two of the nursing homes (*n*_*1*_ = 20; *n*_*2*_ = 10). Others were recruited from the remaining seven nursing homes, with two or three participants residing in each of those nursing homes (*n*_*3-9*_ = 17). A manager of each institution notified the researchers by e-mail when a person arrived in the nursing home. Four days after admission, a first appointment was made with this person to discuss the details of the project. In case the participant agreed to participate, the French version of the Telephone Interview for Cognitive Status Modified (F-TICS-m; Lacoste and Trivalle 2009), was administered face-to-face for the initial cognitive screening. An inclusion cut-off score of 28 and above for the F-TICS-m was used (Vercambre et al. 2010). Respondents were interviewed the first month (T1; *M*_*days*_ = 12.72, *SD*_*days*_ = 4.67), three months (T2; *M*_*days*_ = 105.35, *SD*_*days*_ = 10.37) and six months after relocation (T3; *M*_*days*_ = 195.81, *SD*_*days*_ = 17.78). Respondents completed the Cog-Tel and answered self-administered questionnaires. To avoid response bias due to fatigue, the presentation order of questionnaires was randomized among respondents.

#### Statistical analyses

Repeated measures ANOVAs were used to assess changes in each indicator of cognitive functioning and quality of life between the three time points. Additionally, we explored whether relational resources impacted changes in cognition and quality of life, by including these indicators as between-person factors. In case of significant changes, post-hoc pairwise comparisons were performed with Bonferroni corrections for multiple comparisons (i.e., *p*-value multiplied by the number of comparisons made, indicated as *p*_*adj*_). Alpha-level was set to 0.05.

## Results

### Descriptive analysis

Table 1 displays the descriptive statistics. Full details on Pearson correlations between the variables of interest with age, gender, and education are reported in Supplementary Information S2.

**Table 1:** Descriptive statistics at all assessments (N = 47)

	T1			T2			T3		
	*M*	*SD*	Range	*M*	*SD*	Range	*M*	*SD*	Range
*Cognitive functioning*									
Weighted total score	22.43	5.43	12.60–36.30	21.23	5.67	12.50–34.70	21.81	5.00	11.10–32.40
Short-term memory	2.92	1.38	0–6	2.19	1.38	0–6	2.62	1.24	0–5
Long-term memory	3.04	1.56	0–7	2.21	1.74	0–7	2.55	1.50	0–6
Working memory	3.85	1.84	0–9	4.11	1.80	2–9	3.98	1.58	2–9
Verbal fluency	16.43	6.64	4–34	16.15	6.05	5–33	15.81	6.07	3–30
Inductive reasoning	1.98	1.58	0–8	1.96	1.69	0–8	2.15	1.38	0–7
Prospective memory^a^	0.98	0.15	0–1	1.00	0.00	1–1	0.96	0.20	0–1
*Quality of life*									
Depressive symptoms	4.60	3.39	0–12	4.72	3.13	0–13	5.06	3.26	0–12
Loneliness	0.62	0.45	0–1.73	0.86	0.53	0–1.82	0.76	0.50	0–1.73
Social loneliness	0.63	0.56	0–1.80	0.79	0.64	0–2	0.72	0.57	0–1.8
Emotional loneliness	0.62	0.51	0–1.67	0.91	0.58	0–2	0.80	0.65	0–2
Purpose in life	2.27	0.56	1.14–3.50	2.24	0.54	0.71–3.50	2.22	0.50	1–3.14

Range indicates the minimum and maximum scores on the variables of interest. T1, T2, and T3 refer to the assessments during the first month, and three and six months after relocation, respectively

^a^Prospective memory was only included in the total cognition score, and not examined individually due to high performance (i.e., limited variability) across all assessments

### Cognitive functioning

For the mean scores of the weighted total score of cognitive functioning, results revealed no significant change between the three assessments, *F*(2, 92) = 1.880, *p* = 0.158, *η*_p_^2^ = 0.039.

#### Short-term and long-term memory

**Fig. 1:**
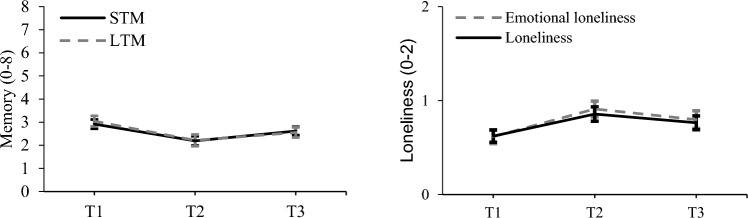
Mean-level changes in short-term and long-term memory, and (emotional) loneliness across the first six months after relocation to a nursing home (N = 47). *Note.* Error bars represent the standard error around the mean for each time point. STM = short-term memory, LTM = long-term memory

Mean levels of short-term memory changed over time (*F*(2, 92) = 5.590, *p* = 0.005, *η*_p_^2^ = 0.108), with pairwise comparisons revealing a significant decline in short-term memory from T1 to T2 (*M*_*diff*_ = 0.72, *SE* = 0.22, *p*_*adj*_ = 0.005). No significant changes were observed between T2 and T3 (*p*_*adj*_ = 0.153) nor between T1 and T3 (*p*_*adj*_ = 0.567). Although non-significant, we descriptively observed a slight increase between T2 and T3, resulting in no significant differences between T1 and T3. Regarding long-term memory, results indicated a similar pattern. The analysis revealed that performance significantly changed over time (*F*(2, 92) = 5.286, *p* = 0.007, *η*_p_^2^ = 0.103), with pairwise comparisons further indicating a significant decline in long-term memory from T1 to T2 (*M*_*diff*_ = 0.83, *SE* = 0.24, *p*_*adj*_ = 0.004). No significant changes were detected from T2 to T3 (*p*_*adj*_ = 0.565), nor from T1 to T3 (*p*_*adj*_ = 0.235). Figure 1 displays the mean-level changes over time for short-term and long-term memory.

#### Working memory, verbal fluency, and inductive reasoning

No significant changes were observed across the three assessments for working memory (*F*(2, 92) = 0.479, *p* = 0.621, *η*_p_^2^ = 0.010), verbal fluency (*F*(2, 92) = 0.421, *p* = 0.658, *η*_p_^2^ = 0.009), and inductive reasoning (*F*(2, 92) = 0.509, *p* = 0.603, *η*_p_^2^ = 0.022).

### Quality of life

#### Loneliness

Regarding loneliness, mean levels changed significantly over time, *F*(2, 92) = 6.671, *p* = 0.002, *η*_p_^2^ = 0.127, with pairwise comparisons revealing a significant increase between T1 and T2 (*M*_*diff*_ = 0.24, *SE* = 0.06, *p*_*adj*_ = 0.001). No differences were detected between T2 and T3 (*p*_*adj*_ = 0.437), nor between T1 and T3 (*p*_*adj*_ = 0.138). Although non-significant, we descriptively observed a slight decrease in loneliness between T2 and T3, leaving no significant differences in mean levels of loneliness between T1 and T3. With regard to the specific subdimensions of loneliness, no significant changes were found for social loneliness (*F*(2, 92) = 1.790, *p* = 0.173, *η*_p_^2^ = 0.037), but results revealed significant changes in emotional loneliness over time, *F*(2, 92) = 6.351, *p* = 0.003, *η*_p_^2^ = 0.121. Pairwise comparisons confirmed an increase in emotional loneliness between T1 and T2 (*M*_*diff*_ = 0.29, *SE* = 0.07, *p*_*adj*_ < 0.001), but no significant changes were detected between T2 and T3 (*p*_*adj*_ = 0.503), nor between T1 and T3 (*p*_*adj*_ = 0.175). Following a similar line of argumentation as discussed for loneliness, these results suggested that emotional loneliness no longer differed from baseline levels at the final assessment. Figure 1 displays the mean-level changes for (emotional) loneliness across the three assessments.

#### Depressive symptoms and purpose in life

Results indicated no significant changes between the first month, three-months, and six-months follow-up for depressive symptoms (*F*(2, 92) = 0.775, *p* = 0.464, *η*_p_^2^ = 0.017) and purpose in life (*F*(2, 92) = 0.221, *p* = 0.802, *η*_p_^2^ = 0.005).

### Relational resources

**Fig. 2:**
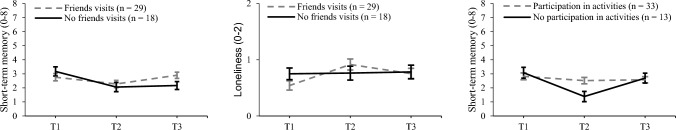
Mean-level changes in short-term memory and loneliness for nursing home residents who were (n = 29) and were not (n = 18) visited by friends during the first month after relocation and mean-level changes in short-term memory for nursing home residents who did (n = 33) and did not (n = 13) participate in activities three months after relocation. *Note.* Error bars represent the standard error around the mean for each time point

In addition to our main analyses, we explored whether relational resources impacted the previously observed changes in short-term and long-term memory, as well as (emotional) loneliness during the first six months after older adults relocated to a nursing home (also see Fig. S1). Only visits by friends at T1 and participation in activities at T2 were found to impact changes in short-term memory and loneliness (for all results see Table S4). Table 2 displays the descriptive information of short-term memory and loneliness at the three assessments for the categories of these between-person factors separately. The main findings are displayed in Fig. 2.

**Table 2:** Descriptive statistics for short-term memory and loneliness reported for the relevant between-person categories, separately

		T1			T2			T3		
Outcome	Between-person category	*M*	*SD*	Range	*M*	*SD*	Range	*M*	*SD*	Range
STM	Visited by friends at T1 (*n* = 29)	2.76	1.41	0–6	2.28	1.62	0–6	2.90	1.11	1–5
	Not visited by friends at T1 (*n* = 18)	3.17	1.34	1–6	2.06	0.87	0–4	2.17	1.34	0–5
	Participated in activities at T2 (*n* = 33)	2.82	1.36	0–6	2.52	1.23	1–6	2.58	1.30	0–5
	Did not participate in activities at T2 (*n* = 13)	3.08	1.50	1–6	1.38	1.50	0–5	2.69	1.18	1–5
Loneliness	Visited by friends at T1 (*n* = 29)	0.54	0.46	0–1.73	0.92	0.54	0–1.82	0.75	0.50	0–1.55
	Not visited by friends at T1 (*n* = 18)	0.75	0.40	0–1.50	0.76	0.50	0.09–1.55	0.78	0.53	0–1.73

One respondent had missing information on participation in activities at T2. STM = short-term memory. T1, T2, and T3 refer to the assessments during the first month, and three and six months after relocation, respectively

#### Visits by friends

In total, 18 older adults (38.3%) were not visited by friends during the first month after relocation. Results suggested that changes in short-term memory were affected by whether or not older adults were visited by friends at T1, *F*(2,90) = 3.418, *p* = 0.037, *η*_p_^2^**=** 0.071. Pairwise comparisons suggested that only individuals who were not visited by friends at T1 experienced a decline in short-term memory between T1 and T2 (*M*_*diff*_ = 1.11, *SE* = 0.35, *p*_*adj*_ = 0.007), and T1 and T3 (*M*_*diff*_ = 1.00, *SE* = 0.34, *p*_*adj*_ = 0.015), while no significant changes were found between T2 and T3 (*p*_*adj*_ > 0.999). These results indicate that individuals who were not visited by friends during the first month after relocation showed a decline in short-term memory and did not recover to baseline levels within six months after relocation. Moreover, six months after relocation, older adults who were not visited by friends during the first month after relocation performed worse on short-term memory compared to those who were visited (T3; *M*_*diff*_ = 0.73, *SE* = 0.36, *p* = 0.049).

Results further suggested that changes in loneliness depended on whether or not older adults were visited by friends at T1, *F*(2, 90) = 3.883, *p* = 0.024, *η*_p_^2^ = 0.079. Pairwise comparisons specifically indicated that only older adults who were visited by friends during the first month after relocation increased in loneliness between the first month and three-months follow-up (*M*_*diff*_ = 0.37, *SE* = 0.07, *p*_*adj*_ < 0.001). Within this group of older adults, no significant changes in loneliness were found between T2 and T3 (*p*_*adj*_ = 0.134), nor between T1 and T3 (*p*_*adj*_ = 0.063). Although non-significant, we descriptively observed a slight decrease in loneliness between T2 and T3, resulting in non-significant differences in loneliness between T1 and T3.

#### Participation in activities

Three months after older adults relocated to a nursing home, 13 individuals (28.3%) reported not to participate in activities at T2. Results suggested that changes in short-term memory were affected by whether or not nursing home residents participated in activities at T2, *F*(2,88) = 5.298, *p* = 0.007, *η*_p_^2^ = 0.107. Pairwise comparisons revealed that only those individuals who did not participate in activities at T2 declined in short-term memory between T1 and T2 (*M*_*diff*_ = 1.69, *SE* = 0.38, *p*_*adj*_ < 0.001) and increased in short-term memory between T2 and T3 (*M*_*diff*_ = 1.31, *SE* = 0.38, *p*_*adj*_ = 0.004). No significant change in short-term memory was found between T1 and T3 (*p*_*adj*_ > 0.999). Specifically, older adults who did not participate in activities three months after relocation declined in short-term memory between the first month and three-months follow-up, and increased back to baseline levels thereafter. Moreover, they performed worse in short-term memory compared to older adults who did participate in activities three months after relocation (T2; *M*_*diff*_ = 1.13, *SE* = 0.43, *p* = 0.011).

## Discussion

This study contributed to previous literature by examining short-term changes in multiple domains of cognitive abilities and quality of life within six months after older adults relocated to a nursing home. Furthermore, exploratory analyses examined the potential role of relational resources in the observed changes in cognitive functioning and quality of life. This study aimed to provide insights into older adults’ adaptive capacities in response to a relocation to a nursing home to further inform theories on processes of successful aging in older age.

We expected cognitive abilities and quality of life to decline after relocation, and further explored whether these changes lasted or were temporary within a six-month follow-up. As hypothesized, we observed a large decline in short-term and long-term memory (*Hypothesis 1*) and a large increase in (emotional) loneliness (*Hypothesis 2*) up to three months after relocation. Following these initial declines, older adults recovered back to baseline levels. Regarding the other indicators of cognitive abilities or quality of life, no changes were found. Exploratory results further revealed a potentially important role of friends’ visits and participation in activities in whether and how older adults changed in short-term memory and loneliness after relocation.

Contrary to previous studies indicating negative long-term impacts of a relocation to a nursing home on cognitive functioning (Harmand et al. 2014; Scocco et al. 2006), the present results indicated a temporary decline in short-term and long-term memory within the three months after relocation before returning to baseline levels. Although these results suggest the declines to be temporary, complementary analyses indicated that specifically older adults who were not visited by friends during the first month after relocation experienced declines in short-term memory, which did not recover up to six months after relocation. These findings are in line with the potential lasting negative impacts of relocation to a nursing home on cognitive functioning which were observed in previous studies (Harmand et al. 2014; Scocco et al. 2006). Moreover, only older adults who did not participate in activities three months after relocation were found to decline in short-term memory performance between the first month and three months after relocation and recovered afterwards. These findings provide preliminary evidence for the importance of relational resources for the maintenance of cognitive functioning within the first months after relocation to a nursing home (Cullati et al. 2018; Kelly et al. 2017).

Regarding quality of life, our study revealed large effects of increased feelings of (emotional) loneliness between the first month and three months after older adults relocated to a nursing home. Similar to the changes observed in memory, these changes were temporary as older adults returned to their baseline levels afterwards. We did not observe any changes in depressive symptoms, purpose in life, or social loneliness after older adults relocated to a nursing home. These findings suggest that older adults may have specifically experienced a lack of emotional connectedness and intimacy within relationships (Cooney 2012). Exploratory analyses further suggested that older adults who were visited by friends during their first month after relocation, experienced increases in loneliness between the first month after relocation and three-months follow-up. No changes were observed for older adults who were not visited by friends. Although seemingly counterintuitive, an explanation could be that friends’ visits decreased in frequency over time (e.g., Port et al. 2001). Older adults who were not visited in the first place, may not have experienced this loss in contact or may have invested more into establishing new social connections, and hence may have not experienced similar increases in loneliness after they relocated. However, experiences of loneliness recovered back to baseline levels, relating to the adaptational capacities of older adults when confronted with major life transitions (Baltes et al. 1991; Cooney 2012; Freund and Baltes 2002; Owen et al. 2023).

The findings of our study indicate that psychosocial adaptational capacities are preserved even in a vulnerable population of nursing home residents. In line with psychosocial models of successful aging (Baltes and Baltes 1990; Freund and Baltes 2000, 2002), we identified successful adaptational patterns in response to the major life transition of a relocation to a nursing home. The observed effect sizes of changes in short-term and long-term memory, and (emotional) loneliness indicate moderate to large effects (Cohen 1988). Although these changes remained short-term, they were substantial in magnitude and suggest important practical implications for the initial adaptation phase after the relocation to a nursing home. Previous findings regarding adaptational patterns after major life transitions highlight that effect sizes vary considerably across studies, even for the same life event (for a meta-analysis, see: Luhmann et al. 2012). For example, the authors reported medium to large effect sizes for change in affective well-being for bereavement and small to medium effect sizes for change in affective well-being for relocation and (voluntary) migration. With regard to the specific life transition of a relocation to a nursing home, little is known about the sizes of reported effects (e.g., Harmand et al. 2014; Scocco et al. 2006). The moderate to large effects found in our study likely reflect the initial impact of a relocation to a nursing home, which may be obscured in studies including longer-term follow-up assessments (Luhmann et al. 2012). Hence, the findings of our study further emphasize the added value of including short-term assessments in order to adequately capture the impact and adaptational patterns following life transitions. Moreover, exploratory findings highlight that the maintenance or re-establishment of relational resources may play a crucial role in this adaptive process after this major life transition. Specifically, regarding short-term memory, relational resources were suggested to facilitate recovery of the initial negative impact or even to prevent an adverse effect altogether.

## Limitations and future studies

Relocation to a nursing home is often prompted by common challenges in older age such as (sudden) health events or functional and cognitive declines (Gaugler et al. 2007). Declines or losses in these domains potentially negatively impact quality of life (Bondevik and Skogstad 2000; Owen et al. 2023; Pfund and Lewis 2020) or the ability of individuals to maintain or re-establish relational resources (Cullati, et al., 2018; Baltes et al. 1991). Since we lacked information on any of the studied indicators before relocation, we must be cautious in generalizing the observed changes as direct consequences of the specific life transition of a relocation to a nursing home. Moreover, measures of cognitive abilities or quality of life may have already been negatively impacted by relocation at the first assessment, limiting our conclusions that older adults were not affected in other domains of cognitive abilities or quality of life, nor that they fully adapted to the transition of short-term and long-term memory or (emotional) loneliness. Additionally, correlations revealed some interdependencies between our primary constructs of interest (i.e., cognitive functioning, quality of life, and relational resources) and potential effects of age, gender, and education (see Supplementary Information S2). Specifically, younger, lower-educated, female nursing home residents seemed to be more at risk of worse outcomes. Future studies could examine interdependencies between cognitive abilities, quality of life, and relational resources, including relevant confounding factors in the context of relocation to a nursing home. 

Relating to the indicators used in this study, there are a few more limitations to mention. First, the indicators of quality of life did not fully capture the multidimensionality of this construct, including, for example, aspects of subjective evaluations of physical health (e.g., mobility), life satisfaction, autonomy, and living conditions (Brazier et al. 1992; Hyde et al. 2003). Moreover, our study lacked information on relational resources before the transition to a nursing home, hindering us from studying changes in these resources over time.

Participants in our study were clustered in different nursing homes, which we were unable to account for. Future studies should replicate our findings in sufficiently large samples to investigate short-term changes in cognition and quality of life after relocation to a nursing home, ideally using cluster-based modeling approaches. Additionally, our study included cognitively intact nursing home residents. Previous research indicated that, specifically, individuals with lower baseline cognitive functioning show steeper declines in cognitive abilities (e.g., Schaeverbeke et al. 2021; Mouchet et al. 2021). As such, the findings of our study may only generalize to older adults who are cognitively fit when entering the nursing home. Similar to previous work (Lucas 2007; Verspeek et al. 2024), our study suggests that individuals differ in how they respond to major life transitions. Hence, average change or stability might mask differential changes observed at the individual level. The current study was underpowered to examine individual differences in change in cognitive abilities or quality of life after the transition to a nursing home. Future studies should examine individual differences in change in a larger, more representative sample of nursing home residents to further investigate short-term adaptational processes following nursing home admission.

## Conclusion

The findings of this study indicated potential short-term consequences for both cognitive functioning and quality of life after a relocation to a nursing home. Although results suggested that individual psychosocial adaptational capacities may be preserved in a highly vulnerable population, individual differences in preservation of such capacities are very likely. Hence, nursing home residents may need continued and proactive support from existing social networks as well as nursing home staff to further facilitate adaptational processes shortly after this major life transition.

## Supplementary Information

Below is the link to the electronic supplementary material.

Supplementary file1 (DOCX 594 KB)

## Data Availability

No datasets were generated or analysed during the current study.
