# Supplementary material for: Changes in cognitive functioning and quality of life after relocation to a nursing home: a prospective longitudinal study of Swiss nursing home residents
Source: Eur J Ageing. 2025 Jul 22;22(1):35. doi: 10.1007/s10433-025-00869-7 (PMC12283509; doi:10.1007/s10433-025-00869-7)
Supplement: Supplementary file 1 — Supplementary file1 (DOCX 594 KB) [file 10433_2025_869_MOESM1_ESM.docx]

**Supplementary Information**

**Changes in Cognitive Functioning and Quality of Life After Relocation to a Nursing Home: A Prospective Longitudinal Study of Swiss Nursing Home Residents**

Emmie A. M. Verspeek^1^, Maximilian Haas^2,3^, Yvonne Brehmer^1,4^, Manon A. van Scheppingen^1^, Nadine Bender^2^, Matthias Kliegel^2,5,6^, Alexandra Hering^2,1,5^

^1^ Department of Developmental Psychology, Tilburg University, Tilburg, Netherlands

^2^ Faculty of Psychology and Educational Sciences, University of Geneva, Geneva, Switzerland

^3^ Faculty of Psychology, UniDistance Suisse, Brig, Switzerland

^4^ Aging Research Center, Karolinska Institutet, Stockholm, Sweden

^5^ Centre for the Interdisciplinary Study of Gerontology and Vulnerability, University of Geneva, Geneva, Switzerland

^6^ LIVES, Overcoming Vulnerability: Life Course Perspective, Swiss National Centre of Competence in Research, Geneva, Switzerland

Correspondence concerning this supplementary appendix should be addressed to A. Hering, Faculty of Psychology and Educational Sciences, University of Geneva, 40, Boulevard du Pont-d’-Arve, 1211, Geneva, Switzerland. E-mail: alexandra.hering@unige.ch

Submitted to European Journal of Ageing

**S1: Attrition and Sample Selection**

The samples in the first, second, and third data collection assessments included 61, 50, and 48 participants, respectively. Figure S2 displays exact information on drop-out and sample selection. Table S1 includes the descriptive statistics of age, F-TICS-m, and years of education for the total-, drop-out, and final sample. Sample selectivity was assessed by calculating the normed differences in these indicators between the final sample (*n* = 47) versus the initial sample (*N* = 61) (*M*_finalsample_– *M*_initialsample_) / *SD*_initialsample_; for a similar approach, see Lindenberger et al., 2002). Older adults who remained in the study were younger (by 0.01 *SD* units), had higher F-TICS-m scores (by 0.08 *SD* units), and had less years of education (by 0.05 *SD* units). By convention, none of these differences qualified as a sample selectivity effect (Cohen, 1977).

| **Table S1** |  |  |  |  |  |  |  |
| --- | --- | --- | --- | --- | --- | --- | --- |
| *Descriptive statistics and sample selectivity* | | | |  |  |  |  |
|  | Total sample (*N* = 61) | | Drop-out sample (*n* = 14) | | Final sample (*n* = 47) | |  |
| Indicator | *M* | *SD* | *M* | *SD* | *M* | *SD* | *SD* unit difference |
| Age | 85.62 | 8.85 | 85.86 | 6.86 | 85.55 | 9.43 | -0.01 |
| F-TICS-m | 31.69 | 3.79 | 30.71 | 3.15 | 31.98 | 3.94 | 0.08 |
| Years of education | 11.28 | 3.68 | 11.93 | 4.07 | 11.09 | 3.59 | -0.05 |
|  | | | | | | | |

**S2: Correlations**

Table S2 displays the correlations among the variables of interest in our study at T1. Significant Pearson correlations were found between depressive symptoms and short-term memory (*r* = .32 , *p* = .027), loneliness (*r* = .57, *p* <.001), social loneliness (*r* = .37, *p* = .010), emotional loneliness (*r* = .58, *p* < .001), and purpose in life (*r* = -.64, *p* <.001). These correlations indicated that more depressive symptoms were associated with higher short-term memory performance, more (emotional and social) loneliness, and lower purpose in life. Moreover, purpose in life was significantly correlated with loneliness (*r* = -.52, *p* <.001), social loneliness (*r* = -.47, *p* < .001), and emotional loneliness (*r* = -.41 , *p* = .004), indicating that higher purpose in life was associated with lower (social and emotional) loneliness. Additionally, working memory and purpose in life were significantly correlated (*r* = .34, *p* = .018), showing that higher working memory performance was associated with higher purpose in life. No significant correlations were found between the indicators of cognitive abilities, quality of life, and the indicators of relational resources (i.e., visits by friends and participation in activities) at T1. However, at T2 participation in activities was significantly correlated with total cognitive functioning (*r* = .29, *p* = .047) and short-term memory (*r =* .37, *p* = .011), feeling integrated with depressive symptoms (*r* = -.38, *p* = .010), and feeling at home with purpose in life (*r* = .36, *p* = .015). Those correlations indicate that individuals who participated in activities at T2 had significantly better global cognitive functioning and short-term memory, individuals who felt integrated reported fewer depressive symptoms, and participants who felt at home reported higher purpose in life, compared to those who did not participate in activities, did not feel integrated and at home, respectively.

Table S3 displays the correlations of the variables of interest with age, gender, and education. Significant Pearson correlations were found between age and depressive symptoms (*r* = -.43, *p* = .002), social loneliness (*r* = -.29, *p* = .048), and purpose in life (*r* = .35, *p* = .017), indicating that older participants experienced less depressive symptoms and social loneliness, and higher purpose in life. Moreover, gender was significantly correlated with total cognitive functioning (*r* = -.33 , *p* = .022), working memory (*r* = -.54, *p* < .001), inductive reasoning (*r* = -.43, *p* = .003), and purpose in life (*r* = -.30, *p* = .038). Female participants performed worse on total cognitive functioning, working memory and inductive reasoning, and experienced lower purpose in life than males. Years of education were significantly correlated with total cognitive functioning (*r* = .29, *p* = .045), working memory (*r* = .44, *p* = .002), and verbal fluency (*r* = .29, *p* = .046), indicating that more years of education was associated with better total cognitive performance, as well as for the subtasks of working memory and verbal fluency. Visits by friends or participation in activities were not significantly correlated with age, gender, or education at T1. At T2 no significant correlations were detected between feeling integrated or at home and age, gender, or education. Participation in activities was, however, significantly associated with years of education (*r*  = .32, *p* = .031), indicating that participants with more years of education were more likely to participate in activities at T2.

| **Table S2** |  |  |  |  |  |  |  |  |  |  |  |  |  |
| --- | --- | --- | --- | --- | --- | --- | --- | --- | --- | --- | --- | --- | --- |
| *Pearson correlations of the variables of interest at T1 (N = 47)* | | | | | | | | | | | | | |
|  | Total Cog | STM | LTM | WM | VF | IR | Dep | Lo | Soc Lo | E Lo | Purpose | Visits fr | Part act |
| Total Cog | 1 |  |  |  |  |  |  |  |  |  |  |  |  |
| STM | .553** | 1 |  |  |  |  |  |  |  |  |  |  |  |
| LTM | .593** | .698** | 1 |  |  |  |  |  |  |  |  |  |  |
| WM | . 512** | -.005 n.s. | -.020 n.s. | 1 |  |  |  |  |  |  |  |  |  |
| VF | .713** | .407** | .397** | .381** | 1 |  |  |  |  |  |  |  |  |
| IR | .732** | .129 n.s. | .168 n.s. | .253 n.s. | .380** | 1 |  |  |  |  |  |  |  |
| Dep | .008 n.s. | .322* | .102 n.s. | -.173 n.s. | .070 n.s. | -.014 n.s. | 1 |  |  |  |  |  |  |
| Lo | .091 n.s. | .153 n.s. | .096 n.s. | -.110 n.s. | .235 n.s. | .144 n.s. | .568** | 1 |  |  |  |  |  |
| Soc Lo | .039 n.s. | -.007 n.s. | .000 n.s. | -.122 n.s. | .244 n.s. | .150 n.s. | .370* | .830** | 1 |  |  |  |  |
| E Lo | .117 n.s. | .258 n.s. | .159 n.s. | -.066 n.s. | .162 n.s. | .098 n.s. | .583** | .860** | .429** | 1 |  |  |  |
| Purpose | .202 n.s. | -.030 n.s. | .114 n.s. | .343* | -.062 n.s. | .090 n.s. | -.637** | -.520** | -.471** | -.413** | 1 |  |  |
| Visits fr | .185 n.s. | -.145 n.s. | -.063 n.s. | .032 n.s. | -.122 n.s. | -.179 n.s. | .049 n.s. | -.229 n.s. | -.241 n.s. | -.151 n.s. | .101 n.s. | 1 |  |
| Part act | .106 n.s. | .009 n.s. | .191 n.s. | -.023 n.s. | .206 n.s. | .071 n.s. | .076 n.s. | -.002 n.s. | -.049 n.s. | .044 n.s. | 004 n.s. | .030 n.s. | 1 |
| *Note.* Total Cog = Weighted total cognitive functioning, STM = short-term memory, LTM = long-term memory, WM = working memory, VF = verbal fluency, IR = inductive reasoning, Dep = depressive symptoms, Lo = loneliness, Soc Lo = social loneliness, E Lo = Emotional loneliness, Visits fr = visits by friends (no = ref), Part act = participation in activities (no = ref), n.s. = not significant, ** correlation is significant at the .01 alpha-level, * correlation is significant at the .05 alpha-level | | | | | | | | | | | | | |

| **Table S3** |  |  |  |
| --- | --- | --- | --- |
| *Pearson correlations of variables of interest with age, gender, education, and relational resources at T1 (N = 47)* | | | |
|  | Age | Gender (male = ref) | Years of education |
| Weighted total cognitive functioning | -.130 n.s. | -.333* | .293* |
| STM | -.227 n.s. | .223 n.s. | .006 n.s. |
| LTM | -.123 n.s. | .178 n.s. | .054 n.s. |
| Working memory | .077 n.s. | -.542** | .443** |
| Verbal fluency | -.284 n.s. | -.255 n.s. | .292* |
| Inductive reasoning | -.012 n.s. | -.425** | .173 n.s. |
| Depressive symptoms | -.433** | .278 n.s. | -.120 n.s. |
| Loneliness | -.285 n.s. | .013 n.s. | .175 n.s. |
| Social loneliness | -.290* | -.008 n.s. | .220 n.s. |
| Emotional loneliness | -.201 n.s. | .029 n.s. | .080 n.s. |
| Purpose | .345* | -.304* | .213 n.s. |
| Visits by friends (no = ref) | -.089 n.s. | .081 n.s. | .130 n.s. |
| Participation in activities (no = ref) | -.198 n.s. | -.069 n.s. | .149 n.s. |
| *Note.*  STM = short-term memory, LTM = long-term memory, n.s. = not significant, ** correlation is significant at the .01 alpha-level, * correlation is significant at the .05 alpha-level | | | |

| **Table S4** |  |  |  |  |  |  |  |  |  |  |  |
| --- | --- | --- | --- | --- | --- | --- | --- | --- | --- | --- | --- |
| *Repeated Measures ANOVA with between-person factors for the non-significant interactions between short-term memory, long-term memory, and (emotional) loneliness with several indicators of relational resources.* | | | | | | | | | | | |
|  |  | T1 | | T2 | | T3 | | Repeated measures ANOVA | | | |
| Outcome | Between-person category | *M* | *SE* | *M* | *SE* | *M* | *SE* | *F* | *df* | *p* |  |
| STM | Participated in activities at T1 (*n* = 27) | 2.93 | 0.27 | 2.41 | 0.26 | 2.78 | 0.24 | 0.637 | 2, 90 | .531 |  |
|  | Did not participate in activities at T1 (*n* = 20) | 2.90 | 0.31 | 1.90 | 0.31 | 2.40 | 0.28 |  |  |  |  |
|  | Felt integrated at T2 (*n* = 33) | 2.67 | 0.24 | 2.21 | 0.25 | 2.67 | 0.22 | 2.493 | 2, 88 | .088 |  |
|  | Did not feel integrated at T2 (*n* = 13) | 3.46 | 0.38 | 2.15 | 0.39 | 2.46 | 0.35 |  |  |  |  |
|  | Felt at home at T2 (*n* = 30) | 2.77 | 0.25 | 2.03 | 0.25 | 2.47 | 0.23 | 0.027 | 2, 88 | .974 |  |
|  | Did not feel at home at T2 (*n* = 16) | 3.13 | 0.35 | 2.50 | 0.35 | 2.88 | 0.31 |  |  |  |  |
| LTM | Visited by friends at T1 (*n* = 29) | 2.97 | 0.29 | 2.24 | 0.33 | 2.83 | 0.27 | 1.613 | 2, 90 | .205 |  |
|  | Not visited by friends at T1 (*n* = 18) | 3.17 | 0.37 | 2.17 | 0.42 | 2.11 | 0.35 |  |  |  |  |
|  | Participated in activities at T1 (*n* = 27) | 3.30 | 0.30 | 2.26 | 0.34 | 2.67 | 0.29 | 0.453 | 2, 90 | .637 |  |
|  | Did not participate in activities at T1 (*n* = 20) | 2.70 | 0.35 | 2.15 | 0.39 | 2.40 | 0.34 |  |  |  |  |
|  | Felt integrated at T2 (*n* = 33) | 3.06 | 0.27 | 2.39 | 0.31 | 2.55 | 0.27 | 0.735 | 2, 88 | .483 |  |
|  | Did not feel integrated at T2 (*n* = 13) | 2.85 | 0.43 | 1.77 | 0.49 | 2.62 | 0.43 |  |  |  |  |
|  | Felt at home at T2 (n = 30) | 2.90 | 0.29 | 2.30 | 0.33 | 2.43 | 0.28 | 0.746 | 2, 88 | .477 |  |
|  | Did not feel at home at T2 (*n* = 16) | 3.19 | 0.39 | 2.06 | 0.45 | 2.81 | 0.38 |  |  |  |  |
|  | Participated in activities at T2 (*n* = 33) | 3.09 | 0.27 | 2.52 | 0.30 | 2.55 | 0.27 | 2.015 | 2, 88 | .139 |  |
|  | Did not participate in activities at T2 (*n* = 13) | 2.77 | 0.43 | 1.46 | 0.48 | 2.62 | 0.43 |  |  |  |  |
| Loneliness | Participated in activities at T1 (*n* = 27) | 0.62 | 0.09 | 0.89 | 0.10 | 0.74 | 0.10 | 0.501 | 2, 90 | .608 |  |
|  | Did not participate in activities at T1 (*n* = 20) | 0.62 | 0.10 | 0.81 | 0.12 | 0.79 | 0.11 |  |  |  |  |
|  | Felt integrated at T2 (*n* = 33) | 0.55 | 0.08 | 0.78 | 0.09 | 0.72 | 0.09 | 0.392 | 2, 88 | .677 |  |
|  | Did not feel integrated at T2 (*n* = 13) | 0.81 | 0.12 | 1.03 | 0.14 | 0.86 | 0.14 |  |  |  |  |
|  | Felt at home at T2 (*n* = 30) | 0.65 | 0.08 | 0.83 | 0.10 | 0.79 | 0.09 | 0.605 | 2, 88 | .548 |  |
|  | Did not feel at home at T2 (*n* = 16) | 0.57 | 0.11 | 0.88 | 0.13 | 0.70 | 0.13 |  |  |  |  |
|  | Participated in activities at T2 (*n* = 33) | 0.64 | 0.08 | 0.80 | 0.09 | 0.71 | 0.09 | 1.935 | 2, 88 | .151 |  |
|  | Did not participate in activities at T2 (*n* = 13) | 0.56 | 0.13 | 0.97 | 0.15 | 0.87 | 0.14 |  |  |  |  |
| Emotional loneliness | Visited by friends at T1 (*n* = 29) | 0.56 | 0.09 | 0.97 | 0.11 | 0.82 | 0.12 | 1.589 | 2, 90 | .210 |  |
|  | Not visited by friends at T1 (*n* = 18) | 0.71 | 0.12 | 0.82 | 0.14 | 0.77 | 0.15 |  |  |  |  |
|  | Participated in activities at T1 (*n* = 27) | 0.64 | 0.10 | 0.94 | 0.11 | 0.77 | 0.13 | 0.456 | 2, 90 | 0.635 |  |
|  | Did not participate in activities at T1 (*n* = 20) | 0.59 | 0.11 | 0.87 | 0.13 | 0.84 | 0.15 |  |  |  |  |
|  | Felt integrated at T2 (*n* = 33) | 0.50 | 0.08 | 0.80 | 0.10 | 0.74 | 0.11 | 1.265 | 2, 88 | .287 |  |
|  | Did not feel integrated at T2 (*n* = 13) | 0.89 | 0.13 | 1.12 | 0.15 | 0.85 | 0.18 |  |  |  |  |
|  | Felt at home at T2 (*n* = 30) | 0.60 | 0.09 | 0.83 | 0.10 | 0.78 | 0.12 | 0.651 | 2, 88 | .524 |  |
|  | Did not feel at home at T2 (*n* = 16) | 0.62 | 0.13 | 1.00 | 0.14 | 0.76 | 0.16 |  |  |  |  |
|  | Participated in activities at T2 (*n* = 33) | 0.62 | 0.09 | 0.85 | 0.10 | 0.73 | 0.11 | 0.725 | 2, 88 | .487 |  |
|  | Did not participate in activities at T2 (*n* = 13) | 0.56 | 0.14 | 0.99 | 0.16 | 0.87 | 0.18 |  |  |  |  |
| *Note.* Visits by family were not included, since only four older adults were not visited by family at T1. | | | | | | | | | | | |

**Fig. S1**


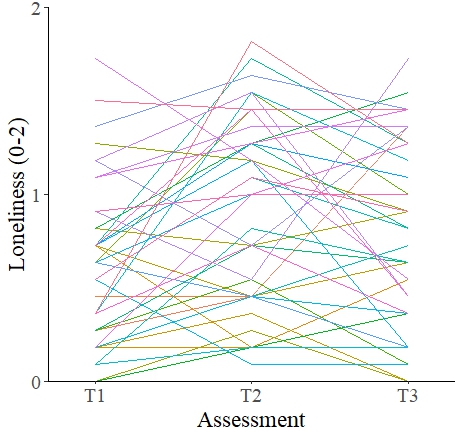

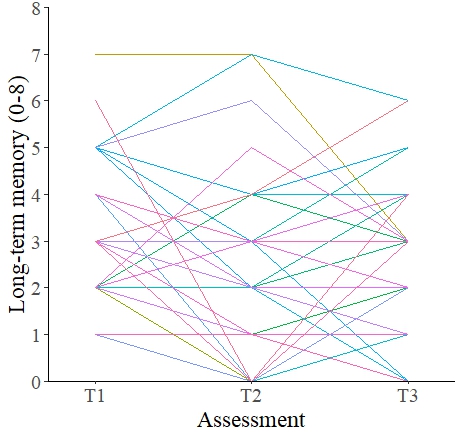

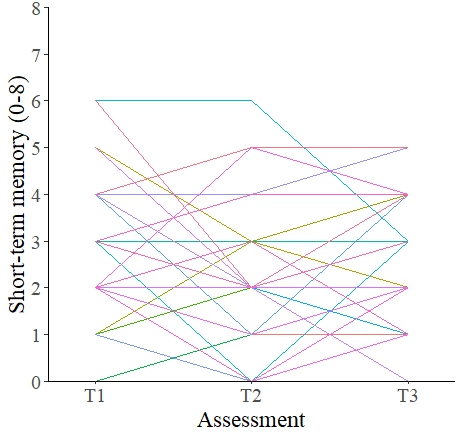

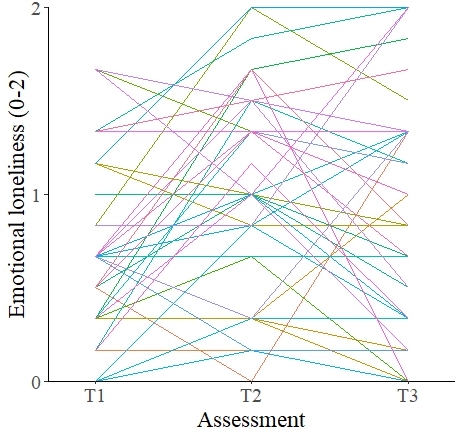
*Individual differences in changes in short-term memory, long-term memory, and (emotional) loneliness (N = 47)*

**Fig. S2**

*Flow chart of sample attrition and selection.*

Nursing home residents contacted (*N =* 110)

First assessment (*n =* 61)

Second assessment (*n =* 50)

Third assessment (*n =* 48)

Did not agree to participate (*n* = 24), not eligible because of cognitive limitations (*n* = 22), hearing loss (*n* = 2), aphasia (*n =* 1)

Returned home (*n =* 1), passed away (*n* =8), unknown reasons for drop-out (*n* = 2)

Passed away (*n* = 1), unknown reasons for drop-out (*n* = 1)

Final sample (*n =* 47)

Excluded because of cognitive limitations (*n =* 1)

**References**

Cohen, J. (1977). *Statistical power analysis for the behavioral sciences* (Rev. ed.). Academic Press.

Lindenberger, U., Singer, T., & Baltes, P. B. (2002). Longitudinal Selectivity in Aging Populations: Separating Mortality-Associated Versus Experimental Components in the Berlin Aging Study (BASE). *The Journals of Gerontology Series B: Psychological Sciences and Social Sciences*, *57*(6), P474–P482. https://doi.org/10.1093/geronb/57.6.P474
